# Fostering Inclusive Curricula and Learning Environments: Inclusivity Reporting at a UK University

**DOI:** 10.1007/s40670-024-02049-1

**Published:** 2024-05-22

**Authors:** Gabrielle M. Finn

**Affiliations:** Faculty of Biology, Medicine and Health, University of Manchester, Manchester, UK

**Keywords:** Inclusion, Diversity, Equality, Medical education, Assessment, Curricula

## Abstract

Universities are under increased scrutiny with respect to creating inclusive curricula and learning environments. Students often worry about reporting a lack of inclusivity, or they simply don't know where to go to do so. The University of Manchester created a rapid inclusivity reporting form, embedded into all programme virtual learning environments to enable accessible, and if preferred anonymous, reporting of issues. The form is triaged, and an accompany communications strategy was developed to ensure the feedback loop to students was complete. The form has been a success, with many issues, particularly related to sex and gender in the curricula being improved.

**Supplementary Information:**

The online version contains supplementary material available at 10.1007/s40670-024-02049-1.

In today’s rapidly evolving world, higher education institutions are often lagging behind societal transformation. Institutions are, rightly, under increased pressure to create inclusive curricula and learning environments. For example, recent events such as the death of George Floyd have resulted in institutions increasing their efforts to decolonize curricula [1]. These initiatives and curriculum transformations matter as the effectiveness and impact of education are intrinsically linked to its inclusivity. Literature proposes comprehensive inclusivity training for faculty [2] and adapting curricula through utilizing inclusive language and resources that negate biases [3]. Inclusive curricula and learning environments are not mere educational buzzwords; they represent the fundamental principles that underscore equitable access, diverse perspectives, and the empowerment of every learner. The sticky issue for institutions is that often we do not know what excludes learners; faculty have blind spots. With inclusivity being part of regulatory requirements, it was critical that we address issues, and ensure student voice was central to any initiatives. As Lawrie et al. state, pedagogies should meet the diversity of learners’ needs, and should not create barriers for particular students or student groups [4]. There are many barriers and enablers to inclusive education, one of which is student voice not being heard [5]. It is for this reason that creating an inclusive learning environment is an institutional priority at the University of Manchester (UoM).

The UoM sought to address this issue within the Faculty of Biology, Medicine, and Health, where there are more than 11,000 undergraduate and postgraduate learners, on over 100 courses. Some courses are regulated by professional bodies including, but not limited to, nursing, midwifery, pharmacy, medicine, and dentistry. An inclusivity reporting initiative was launched in 2022. This initiative enables students to report areas of curricula or elements of student experience that either lack inclusivity or are shining examples of inclusivity. Using a form that has been embedded into virtual learning environment of every programme, students can report issues quickly and easily. Students are also provided with a direct link to the form that can be accessed out with the virtual learning environment.

The form, available as supplementary material (Supplementary Information), enables staff and students to let us know of any issues in their curriculum or assessments which may have cultural implications, be triggering, sensitive, or unfair. These could include references, reading lists, physical resources, scenarios, or assessment items. These issues might be related to accessibility, socio-cultural context, lack of representation, or values. Examples could include the presence of stereotypes, language that is not inclusive, items that assume local or cultural knowledge, limited or no diversity in reading lists or course materials, or activity that excludes particular groups. Users are also advised that they may want to let us know when there is an opportunity to celebrate contributions from underrepresented groups. Users are also encouraged to suggest a solution to their issue or highlight good practice from elsewhere that we should consider adopting.

When issues are reported, they are discussed with the relevant teams to consider what actions might be appropriate. We take great care to inform users that the form does not supersede our complaints or appeals procedure. It is a reporting system which will help us better understand the content of our teaching and make changes where appropriate. When a student raises an issue, it will come through to a Faculty email inbox. The Vice Dean for Teaching triages the response, sending through to the most appropriate person in the School or Programme. Some issues will require action, others will not. Where action is not appropriate, a response as to why will be provided to the student. We log responses so that we can audit, evaluate, and review. We encourage anyone submitting the form to provide their name and email so that we can reply to them with a summary of any action taken, but anonymity is permitted. The entire student body also receives an email from the programme lead to explain the issue raised and the decisions. All staff have received guidance regarding the form, its purpose, and examples of submissions and responses. A workflow is provided in Fig. 1.

**Fig. 1:**
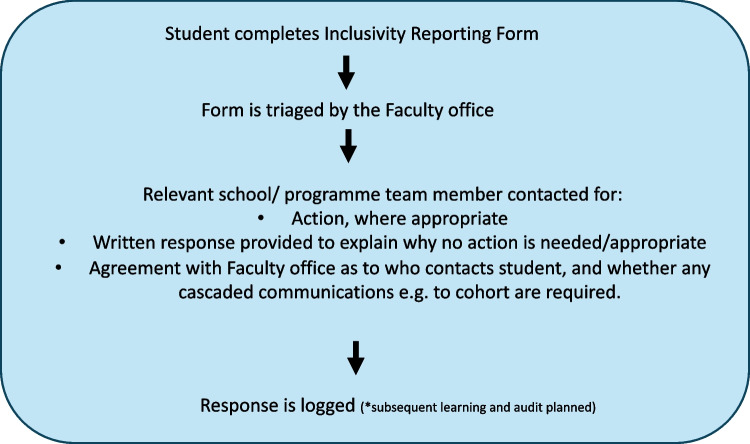
Workflow

The form has been well received by students. Table 1 summarises some recent submissions and the actions taken, under headings taken directly from the form. From the submissions over the 18 months that the form has been active for, it has become evident that sex, sexuality, and gender are problematic areas of the curricula for students. Faculty names and unit codes have been redacted.

**Table 1:** Examples of inclusivity reporting

**Programme**	**What is the material you would like to report?**	**In what way is this material not inclusive?**	**How could we make this more inclusive? Feel free to highlight/link to any good practice examples from elsewhere**	**Response/action**
Biology	Problematic exam timings for students with disabilities	The exams have to be started at 9:45 am, meaning if you have a sleep disorder it may not be the best, or with ADHD your circadian rhythm is different making you most productive in the afternoon/evening. Even without a disorder or disability, it is quite unfair to people who are more productive in the afternoon or people who struggle to wake up early and it feels disadvantageous to struggle with an exam based on being tired	Could give a larger timeframe to do the exam in, e.g., with [unit code] we have an 8-h timeframe to complete the exam. This means there is more a chance for everyone to do the exam when they feel most productive and energetic instead of getting up early and being tired and possibly slower than they otherwise would've been	Exploration with disability support teams, and assessment coordinators to consider how access can be improved
Dentistry	Timetables too last minute and too many changes	Does not include students who gave to plan their commute, student who work part time, students who having caring responsibilities, students who gave anxiety or other mental health conditions	Decentralise timetables and local leads have more control over timetable as they can anticipate problems	Timetable discussed with Faculty leadership. Communication sent to all students to contextualize and explain issues. Student issues raised with Student Experience and Inclusivity leads
Medicine	Teaching on eating disorders, specifically anorexia, is troubling for students who suffer with, or are recovering from, an eating disorder	This content is traumatic for students who suffer with eating disorders	Remove the teaching or make it optional	A content/tigger warning was added to lecture content and associated recordings. Student support services were signposted for individuals effected. The need for the content, and the specific intended learning outcomes, were reiterated
Pharmacy	(1) When mentioning patients or students, using terms like "ladies" "women" "she or he" (2) When covering topics surrounding menstruation, saying women only experience it	Not everyone who outwardly presents a certain way identifies as a woman or a man. there are many different gender identities so referring to people by terms that don't apply to them is insensitive. Also saying she or he is not inclusive as people may use they/them pronouns or neopronouns not everyone who menstruates identifies as a woman	1) Instead of saying "group of ladies", "the gentleman at the back" etc., just say student person pupil and maybe describe an object they're wearing or using if you want to be more specific 2) Just use they/them as it is more inclusive e.g. "if a patient visits the pharmacy, they may be collecting a prescription on someone else's behalf" instead of "if a patient visits the pharmacy, she or he may be collecting a prescription.." 3) Say "people who menstruate" instead to be more inclusive	Given there were several submissions reporting issues with the lack of inclusive language, Faculty guides on (1) inclusive curricula, (2) inclusive assessment, (3) pronouns, were recirculated to colleagues All students were sent a welcome week email with information on inclusivity, as well as signposting the inclusivity reporting form Finally, a statement on inclusive language was produced in consultation with the LGBT Society and the Students’ Union. The statement explored the issues in the literature with sex and gender often being conflated, how the lexicon of the healthcare professions field remains predominantly biological and fails to keep pace with societal views and individuals’ identities
Midwifery	This unit was outstanding in highlighting the inequalities faced within healthcare. The guest speakers we had and the safe space to discuss openly questions on LGBTQ + and ethnic minorities really empowered myself and the cohort to be inclusive and how to provide better care. As a student rep I heard a lot of positive responses which I fed back to [the faculty]. I have been challenging myself further by taking part in other workshops such as [name’s] LGBTQ + Competency in Lactation workshop. My aim is to continue challenging myself to ensure that everything and more that I have learnt from this unit spreads throughout my degree and beyond	I believe the course itself is heavily heteronormative. It is very easy to slip back into using 'woman/mother dad/father'. Although some lecturers do use other inclusive terms I do recall [unit number] as an example the term woman was used and we didn't hear other inclusive language. Lectures on vaginal examinations for instance never mentioned about sensitivity and language for trans birthing people. Our assignment title for [unit number] was "Discuss the contemporary role of the midwife in relation to supporting women's choice in the place of birth" Our assignment for unit number] was "Postnatal care for a mother-baby pair who are breastfeeding" and [unit number] is "Evidenced based care plan of midwifery care provided to a woman in preterm labour: "	Whilst I appreciate most service users are women it becomes easy to assume they are addressed as such. Some of the assignments give a choice of scenario to write about such as [unit code]—which are all women scenarios and could be harmful if we assume they were addressed as such. could we perhaps have a trans or non-binary person who wants to be addressed differently. The 6C's are brought up consistently through the course and in particular communication which we write about as a big part of the role. Nursing and Midwifery Council Code 7.3 States to use a range of verbal and non-verbal communication considering cultural sensitivities to better understand and respond to people's person and health needs"—It wouldn't be difficult to write how you would address these needs within a scenario in a few sentences. I know we study breastfeeding more throughout the rest of the course but I'm unsure if any of it covers inducing lactation from birthing partners. I learnt a lot about it on the LGBTQ + lactation workshop which gave me a whole other insight into infant feeding. I do hope this is covered. Another suggestion I had was as part of the core skills we complete each year before going into practice. I think it may be a good idea if we could have a mini core skill/ e-learning resource to remind us about inclusivity each year although I would much rather inclusivity be entwined throughout the course units	

One area that we have worked hard to develop is closing the feedback loop with students. We have achieved this in two ways. Firstly, we host Student Townhall events where the issues raised on the inclusivity forms are tabled as agenda items. Secondly, we have an active ‘You Said, We Did’ campaign. These announcements to students ensure we are accountable and communicate where change has been made, or why we cannot make a change in response to a specific issue. For example, some students raised concerns, based on their religious objections, about transgender health being taught. In this case, inclusion in the curriculum is essential so we did not remove the teaching. Instead, we issued a sensitivity warning to students.

The form has been well received by students and faculty. A testament to this was in the qualitative section of a national student survey where students stated that we had ‘created an environment where everyone belongs and can be themselves’. A Director of Education within a school stated that, “The inclusively reporting form is helpful because when we design and deliver teaching, learning and assessment, whilst we try to do so in an inclusive way, we do not always know how this is received and understood by our diverse student body. The form allows staff to see their teaching from the perspective of the received message which is powerful in understanding where we need to develop our teaching practices to be truly inclusive of those we teach.” Students reported that, “The inclusivity reporting form has given us students a direct channel to teaching leadership to let them know about specific instances when programme content or process does not feel inclusive.” Senior leaders have said that, “the developing log contributes to wider understanding of burning issues and hotspots across our diverse study body and portfolio of programmes. We also hope to provide opportunity for students to feedback when inclusion is being done well, so we can help colleagues and programmes lead by example.”

In sum, we hope that ensuring that inclusivity is at the forefront, that raising issues is easy and accessible, and that solutions are both transparent and clearly communicated will improve the student experience in our institution. Further, this matters for health inequities, as our current students are our future policy makers. Ensuring inclusivity is respected, on their radar, will assist with tackling the troubling inequities in society. We need a diverse workforce to care for a diverse society.

### Supplementary Information

Below is the link to the electronic supplementary material.

Supplementary file1 (PDF 155 KB)
