# Supplementary material for: Fostering Inclusive Curricula and Learning Environments: Inclusivity Reporting at a UK University
Source: Med Sci Educ. 2024 May 22;34(4):731–5. doi: 10.1007/s40670-024-02049-1 (PMC11296976; doi:10.1007/s40670-024-02049-1)
Supplement: Supplementary file 1 — Supplementary file1 (PDF 155 KB) [file 40670_2024_2049_MOESM1_ESM.pdf]

MANCHESTER 1824
The University of Manchester

Inclusive culture in the curriculum

This form enables staff and students to let us know of any issues in your curriculum or assessments which may have cultural implications, be triggering, sensitive, or unfair. These could include references, reading lists, physical resources, scenarios, or assessment items. These issues might be related to accessibility, socio-cultural context, lack of representation or values. Examples could include the presence of stereotypes, language that is not inclusive, items that assume local or cultural knowledge, limited or no diversity in reading lists or course materials, or activity that excludes particular groups. You may want to let us know when there is an opportunity to celebrate contributions from underrepresented groups.

When issues are reported, they will be discussed with the relevant teams to consider what actions might be appropriate. This is not a complaints or appeals procedure. It's a reporting system which will help us better understand the content of our teaching and make changes where appropriate.

Please provide details of the issue below. You can complete this form anonymously if you prefer. To help us take action quickly, it would be helpful if you could please provide unit names and codes, assessment dates or paper references. Thank you in advance for sharing your feedback, and helping us improve.

Programme Title:

Year:

Unit Code:

Assessment or session date (if applicable):

What is the material you would like to report?

In what way is this material not inclusive?

How could we make this more inclusive? Feel free to highlight/link to any good practice examples from elsewhere

If you wish to be updated on the issues raised please provide your details below.

Name:

Student ID or Email Address:

For any support, please contact Professor Gabrielle Finn gabrielle.finn@manchester.ac.uk
